# Development of a Smartphone-Based Nanozyme-Linked Immunosorbent Assay for Quantitative Detection of SARS-CoV-2 Nucleocapsid Phosphoprotein in Blood

**DOI:** 10.3389/fmicb.2021.692831

**Published:** 2021-08-23

**Authors:** Bochao Liu, Ze Wu, Chaolan Liang, Jinhui Lu, Jinfeng Li, Ling Zhang, Tingting Li, Wei Zhao, Yongshui Fu, Shuiping Hou, Xi Tang, Chengyao Li

**Affiliations:** ^1^Department of Transfusion Medicine, School of Laboratory Medicine and Biotechnology, Southern Medical University, Guangzhou, China; ^2^Guangzhou Blood Center, Guangzhou, China; ^3^Shenzhen Key Laboratory of Molecular Epidemiology, Shenzhen Center for Disease Control and Prevention, Shenzhen, China; ^4^Laboratory of Biosafety, School of Public Health, Southern Medical University, Guangzhou, China; ^5^Microbiological Laboratory, Guangzhou Center for Disease Control and Prevention, Guangzhou, China; ^6^Department of Infection, The First People’s Hospital of Foshan, Foshan, China

**Keywords:** SARS-CoV-2, nucleocapsid phosphoprotein, Pt@AuNPs, POCT device, smartphone

## Abstract

Since December 2019, a novel coronavirus (SARS-CoV-2) has resulted in a global pandemic of coronavirus disease (COVID-19). Although viral nucleic acid test (NAT) has been applied predominantly to detect SARS-CoV-2 RNA for confirmation diagnosis of COVID-19, an urgent need for alternative, rapid, and sensitive immunoassays is required for primary screening of virus. In this study, we developed a smartphone-based nanozyme-linked immunosorbent assay (SP-NLISA) for detecting the specific nucleocapsid phosphoprotein (NP) of SARS-CoV-2 in 37 serum samples from 20 COVID-19 patients who were diagnosed by NAT previously. By using SP-NLISA, 28/37 (75.7%) serum samples were detected for NP antigens and no cross-reactivity with blood donors’ control samples collected from different areas of China. In a control assay using the conventional enzyme-linked immunosorbent assay (ELISA), only 7/37 (18.91%) serum samples were detected for NP antigens and no cross-reactivity with control samples. SP-NLISA could be used for rapid detection of SARS-CoV-2 NP antigen in primary screening of SARS-CoV-2 infected individuals.

## Introduction

By the middle of December 2019, the first discovery of unexplained pneumonia was reported in Wuhan, China. It showed person-to-person transmission and was highly contagious during the incubation period (Li Q. et al., 2020; Wang et al., 2020). On January 8, 2020, the new coronavirus was initially confirmed as the pathogen of the outbreak and was declared as “SARS-CoV-2” by the International Committee for Classification of Viruses (Chen N. et al., 2020; Cheng and Shan, 2020; Zhou et al., 2020). The spike protein of SARS-CoV-2 is one of the main proteins for vaccine development, and the nucleocapsid phosphoprotein (NP) encapsulates the viral genome and can be used as a diagnostic antigen (Chan et al., 2020; Chu et al., 2020; Phelan et al., 2020; Wu et al., 2020).

Through viral nucleic acid test (NAT), nearly 170 million people were diagnosed with SARS-CoV-2 infection in the middle of June 2021. Globally, more than three million deaths were announced, and among them more than 5,000 deaths were Chinese. In the diagnosis of COVID-19, the detection of viral nucleic acids has now become a gold standard for SARS-CoV-2 infection (Shen et al., 2020; Yan et al., 2020). The process of viral nucleic acid detection usually requires sampling of nasal, pharyngeal, or anal swabs, which requires a special laboratory environment, testing personnel, and instruments (Peng et al., 2020). If the diagnosis is based solely on viral nucleic acid detection, the workload will be too heavy as there are a large number of suspected cases for testing. Due to uncontrolled factors during sample collection or storage, a false negative or positive results may occur (Tanner et al., 2015; Lim et al., 2018; Kim et al., 2019; Patra et al., 2019; Corman et al., 2020; Zhang et al., 2020).

The detection of anti-SARS-CoV-2 IgM/IgG in the serum is useful as part of the diagnostic process during the pandemic period (Li Z. et al., 2020). However, specific antibody examination for conferring an infection requires 7 days or longer (Xiao et al., 2020; Zhao et al., 2020), and this may be more complex in populations immunized with COVID-19 vaccines; this makes it difficult to determine the infections.

In contrast to the viral nucleic acid and antibody tests, the antigen test can detect the virus itself and facilitate the large-scale screening of crowds. Though some testing of specific antigens of SARS-CoV-2 has recently been commercially available, the main methods have been the enzyme-linked immunosorbent assay (ELISA) and colloidal gold test strips (Chen Z. et al., 2020; Lambert-Niclot et al., 2020; Moitra et al., 2020; Porte et al., 2020). Both of the methods had shortcomings: the ELISA was time consuming with a need for a microplate reader, while the results of the colloidal gold test strip were judged by the naked eye with low sensitivity. Thus, there is still a need for rapid and sensitive testing methods.

In enzyme immunoassays (EIAs), there are several critical limitations for the application of natural enzymes, such as being unstable in harsh conditions, such as different temperatures or pH, and also expensive costs for purification or storage (Rashidian et al., 2013). By comparison with natural enzymes, nanomaterial-based artificial enzymes (nanozymes) have advantages in terms of their stability, cost, and large surfaces for bio-conjugation (Lin et al., 2014). As the most common method for virus detection, ELISA requires specialized training and expensive instruments. Therefore, low-cost and accurate devices are urgently needed for point-of-care testing (POCT).

In this study, we designed a smartphone-based nanozyme-linked immunosorbent assay (SP-NLISA) for detection of SARS-CoV-2 NP antigens, which has several innovative advantages. Firstly, the results can be read by a self-produced POCT device, which is cost-effective and easy to operate. This method thus does not need a well-trained technician and can be used for self-checks at home. Secondly, the results can be sent to a smartphone *via* Bluetooth, which makes data transmission more efficient. Thirdly, the procedure of this assay saves time. Compared with the traditional ELISA method (1–2 h), the testing of this assay can be carried out within 1 h. Finally, this assay is sensitive, reporting results as low as 10 pg/mL of the NP antigen. Thus, this assay is simple, sensitive, rapid, and cost-effective for NP antigen detection in SARS-CoV-2-infected individuals.

## Materials and Methods

### Blood Specimens

In total, 37 serum samples from 20 COVID-19 patients were provided by the Shenzhen Center for Disease Control and Prevention (CDC), and these tested positive for SARS-CoV-2 RNA by real-time RT-PCR (RT-qPCR). A total of 450 negative control samples prior to the COVID-19 outbreak were collected from healthy blood donors by 4 Chinese blood centers: Xi’an (northwest), Harbin (northeast), Guangzhou (south), and Chengdu (southwest). This study was approved by the Medical Ethics Committees of Southern Medical University (SMU) and Shenzhen CDC and followed the ethical guidelines of the 1975 Declaration of Helsinki.

### Chemicals and Reagents

Chloroauric acid (HAuCl_4_⋅3H_2_O), chloroplatinic acid (H_2_PtCl_6_⋅6H_2_O), trisodium citrate, and bovine serum albumin (BSA) were purchased from Sigma-Aldrich (St. Louis, MI, United States). Tween-20, horseradish peroxidase (HRP), hydrogen peroxide (30% H_2_O_2_), and tetramethylbenzidine (TMB) were purchased from Macklin (Shanghai, China). The magnetic beads (MBs), with a diameter of 5 μm, were purchased from Beaverbio (Suzhou, China). The reference standard of SARS-CoV-2 NP antigen was purchased from Bioeast Biotech (Hangzhou, China). The NP (SARS-CoV-2) Detection ELISA Kit was purchased from Biodragon Immunotechnologies (Beijing, China).

### Design of the Smartphone-Based Device

The smartphone-based device for the nanozyme-linked immunosorbent assay (NLISA) was designed to generate colorimetric signals and to export results. This smartphone-based device (SP-device) contained two parts: an optical reader and a photometer. The inside structure of the optical reader was composed of light source (power laser emitter with excitation wavelength 655 nm), light source adjustment, battery, USB charging, and a bracket to place the microwells (Supplementary Figure 1A). A 3D printer was used to fabricate the external structure of this optical reader (Supplementary Figure 1B). The photometer was purchased from Uni-Trend Technology Co., Ltd. (Shenzhen, China) (Supplementary Figure 1C). After the light signal was received by the light sensor of the photometer, it was transmitted and converted into the digital reading. The device was connected to the smartphone *via* Bluetooth and the final result was exported and read in the iENV app. The cost of this device was listed in Supplementary Table 1, conferring an extremely low cost of use.

### Production of Monoclonal Antibodies to SARS-CoV-2 NP

The monoclonal antibodies (mAbs) to NP were produced according to previously reported methods (Li et al., 2017; Liu et al., 2019). Briefly, the three BALB/c mice were immunized by intraperitoneal injection with 25 μL of 1 mg/mL SARS-CoV-2 NP emulsified with an equivalent volume of Freund’s complete adjuvant, and after 2 weeks, a dose of immunogen was intraperitoneally administered using Freund’s incomplete adjuvant. In total, 3 days after the booster injection, the spleen cells of sacrificed mice were fused with murine myeloma SP2/0 cells using 50% polyethylene glycol 4000. After 12 days, the supernatants from hybridomas growing wells were screened using an indirect ELISA. Hybridomas showing positive results were cloned three times by the limiting dilution method, and the mAbs were characterized by ELISA.

### Preparation of Pt@AuNPs

As previously reported, the gold nanoparticles (AuNPs) were synthesized with slight modifications (Wang et al., 2006; Liu et al., 2017). Typically, 1 mL of 1% HAuCl_4_ solution was added into 95 mL of heated deionized water. Then, 4 mL of 1% trisodium citrate were added and stirred for over 10 min after boiling, the color of which would become wine-red during this period. Then, the Pt-Au (shell-core) nanoparticles (Pt@AuNPs) were synthesized by adding 597 μL of 20 mM H_2_PtCl_6_ to 8.403 mL AuNPs. Eight hundred microliters of ascorbic acid (10 mM) were slowly added into the mixture after being heating to 90°C and stirred for 30 min. The obtained Pt@AuNPs had diameters of approximately 40 nm *via* a transmission electron microscope (TEM).

### Preparation of mAb1-Pt@AuNPs and mAb2-MBs

In total, 20 μL of 1 mg/mL mAb1 (clone 5E4) which was used as detecting antibody was added into 1 mL of synthesized Pt@AuNPs. After 1 h, 100 μL of blocking buffer were added and kept for 30 min. Then, after 5 min centrifugation, the unconjugated mAb1 was removed and 100 μL of sample buffer (PBST + 1% BSA) was added to suspend the mAb1-Pt@AuNPs conjugates. Twenty five microliters of MBs were washed with 100 mM 4-Morpholineethanesulfonic acid hydrate buffer (MES) and suspended in 500 μL of phosphate buffered saline (PBS). Then, 2.5 μL each of 1-(3-Dimethylaminopropyl)-3-ethylcarbodiimide hydrochloride (EDC) (1 mg/mL) and N-Hydroxysuccinimide (NHS) (2 mg/mL) were added and reacted for 1 h. The excessive EDC and NHS were removed by centrifugation. In total, 20 μL of 1 mg/mL of mAb2 (clone 4G11) was then added, and the mixture was reacted for an additional 1 h. Finally, 50 μL of 10% BSA were added to block the surface of MBs, and the mAb2-MBs were suspended in 250 μL of PBS and stored at 4°C.

### The Procedure of Pt@AuNPs or HRP Based ELISA

The microwells (Corning, United States) were coated overnight with 5 μg/mL of mAb2 specific to SARS-CoV-2 NP (clone 4G11). Diluted NP antigen standards or serum samples were added and incubated for 90 min. After being washed three times, we added 5 μL of Pt@AuNPs or HRP labeled as 5E4 (mAb1), incubating this for 40 min. Finally, the substrate solution (50% TMB + 50% H_2_O_2_) was added and catalyzed for 10 min. The cut-off value was set as a mean value of negative control plus 2 SD. The limit of detection (LOD) was defined as the lowest level of the NP standard that was tested higher than the cut-off value.

### The Procedure of SP-NLISA for SARS-CoV-2 NP Testing

In total, 100 μL of different concentrations of NP or serum samples (diluted with 0.01 M PBS containing 0.5% Tween, 1% BSA, and 0.01% casein) were added into the freeze-dried microwells contained with a volume of mAb2-MBs and mAb1-Pt@AuNPs. The mixture was reacted for 40 min, and the supernatant was removed by magnets. After washing twice, the microwells were incubated with 100 μL of substrate solution (50% TMB + 50% H_2_O_2_) for 10 min. Finally, the results were determined by the smartphone-based device.

### Statistical Analysis

All experiments were performed three times independently. The data of results were analyzed using the statistical package SPSS v. 16.0 and presented as the mean ± SD. The method of Student’s *t*-test was used to analyze the difference between groups, and a *P*-value < 0.05 was considered statistically significant.

## Results

### The Working Principle of SP-NLISA

**FIGURE 1:**
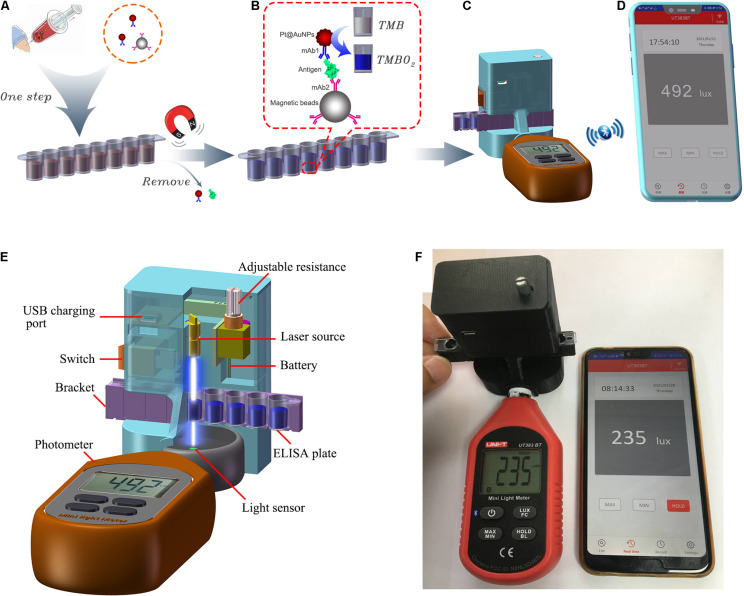
The schematic diagram of SP-NLISA. **(A)** The serum sample (1:1 diluted with sample buffer) was aliquoted into the freeze-dried microwells and mixed for 40 min at 37°C. **(B)** The magnets were used to capture the complexes after washing and the substrate solution (50% TMB + 50% H_2_O_2_) was catalyzed by Pt@AuNPs in the complexes for 10 min at 37°C. **(C)** The microwells were located in the smartphone-based device and **(D)** the results were shown using the iENV app. **(E)** The main structure of the smartphone-based device for NLISA and **(F)** its connection with the app (iENV) in the smartphone *via* Bluetooth.

The main structures and working principles of the SP-NLISA are displayed in Figure 1. In a typical assay, as shown in Figure 1A, 100 μL of different concentrations of NP standards or clinical samples (double dilution) were added into the microwells with the determined volumes of mAb1-Pt@AuNPs and mAb2-MB to form complexes. The mixture was reacted for 40 min, and the supernatant was removed by magnets. The complexes were re-suspended in 100 μL substrate solution (50% TMB + 50% H_2_O_2_), and the Pt@AuNPs in the complexes catalyzed the substrate solution for 10 min (Figure 1B).

When the light emitted by the laser passed through the solution, the intensity of absorbed light was proportional to the amount of Pt@AuNPs bound to the complexes, which could be received by using the smartphone-based device (Figure 1C). The results were sent to the smartphone app (iENV) *via* Bluetooth, which was used for luminosity measurement and calculation (Figure 1D). The results could be shown by both the device (the operator could check the results in the biological safety cabinet) and the smartphone (the results could be recorded outside the biological safety cabinet to avoid possible aerosol contamination). The main structure of the SP device is shown in Figures 1E,F. The instructions for how to use this app are shown in Supplementary Figure 2. The whole detection process could be carried out within 1 h. As Pt@AuNPs were used in the SP-NLISA as catalysts instead of catalytic enzymes in the traditional ELISA, we defined this method as a nanozyme-linked immunosorbent assay (SP-NLISA).

**FIGURE 2:**
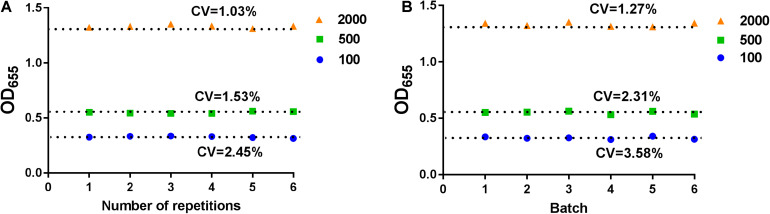
The stability analysis for SP-device. The intra-batch **(A)** or inter-batch **(B)** analysis for SP-device. The low, medium, and high concentrations of NP in normal serum samples were measured by SP-device and the results were converted into OD_655_ values.

To ensure the repeatability of this assay, we tested the stability of this device. The low, medium, and high concentrations of NP in negative serum samples were measured by this SP device. The samples were detected six times in the same batch of tests (Figure 2A) and for six different batches (Figure 2B). The coefficient of variation (CV) of intra-batch or inter-batch was <5%, which showed that the SP device exhibited high stability.

### Characterization of Monoclonal Antibodies to SARS-CoV-2 NP

A total of 34 mAbs reactive with SARS-CoV-2 NP were selected by screening of hybridomas with ELISA. The mAbs were purified from ascitic fluids, and clone 5E4 (mAb1) and clone 4G11 (mAb2) were selected as a pair of specific detection antibodies for SP-NLISA. The titers of the purified ascites fluids of mAbs were 1:1 × 10^6^ (4G11) and 1:2 × 10^6^ (5E4) (Supplementary Figure 3), respectively. The subclass of both mAbs was identified as IgG1. The results demonstrated that these two mAbs had an extremely strong binding capacity to SARS-CoV-2 NP.

### Optimization of SP-NLISA

**FIGURE 3:**
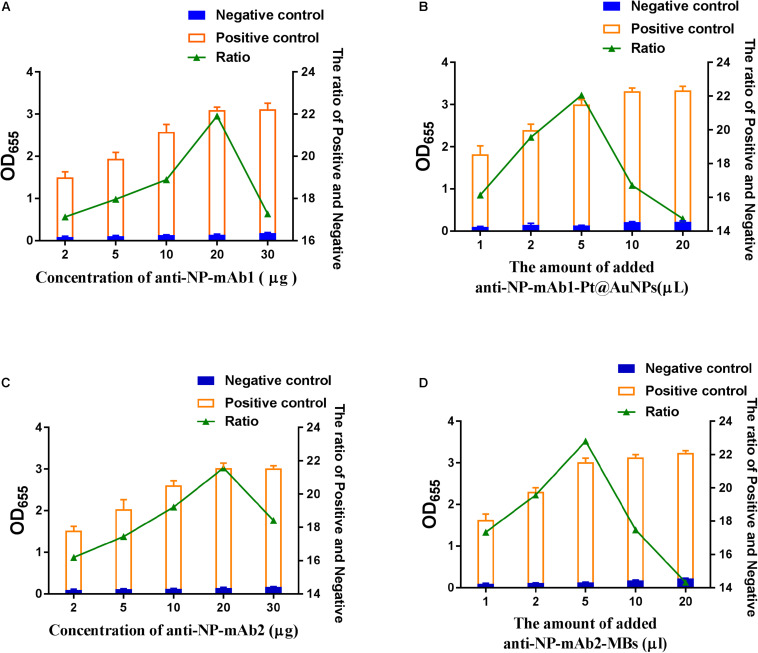
The optimization of SP-NLISA. **(A)** The amounts of monoclonal antibodies (mAb1) labeled Pt@AuNPs were optimized. **(B)** The amounts of the mAb1-Pt@AuNPs were optimized. **(C)** The amounts of monoclonal antibodies (mAbs) labeled MBs were optimized. **(D)** The amounts of mAb2-MBs were optimized. A sample from a healthy blood donor was used as the negative control, and the addition of 1 μg/mL NP standard in the negative control sample was used as the positive control.

We optimized the number of various antibodies and conjugates in this assay. Within the range of 2–30 μg mAb1, 20 μL of 1 mg/mL mAb1 were selected for conjugating with 1 mL Pt@AuNPs, which presented the highest ratio of positivity to negativity (Figure 3A). Furtherly, 5 μL of mAb1-Pt@AuNPs conjugates were determined for use in SP-NLISA (Figure 3B). Similarly, 20 μL of 1 mg/mL mAb2 were selected for conjugating with 25 μL MBs (Figure 3C), and 5 μL of mAb2-MBs conjugates were determined for use in the SP-NLISA (Figure 3D). The optimization of Pt@AuNPs is shown in Supplementary Figures 4, 5. To reduce non-specific reactions, the PBST blocking buffer containing 1% BSA and 0.1% casein was chosen for this assay (Supplementary Figure 6). Finally, the established SP-NLISA was further examined for detection of NP antigen (1 μg/mL) at different storage times and temperatures (Supplementary Figures 7A,B), showing that this system was functionally stable.

### Measurement of NP by SP-NLISA

**FIGURE 4:**
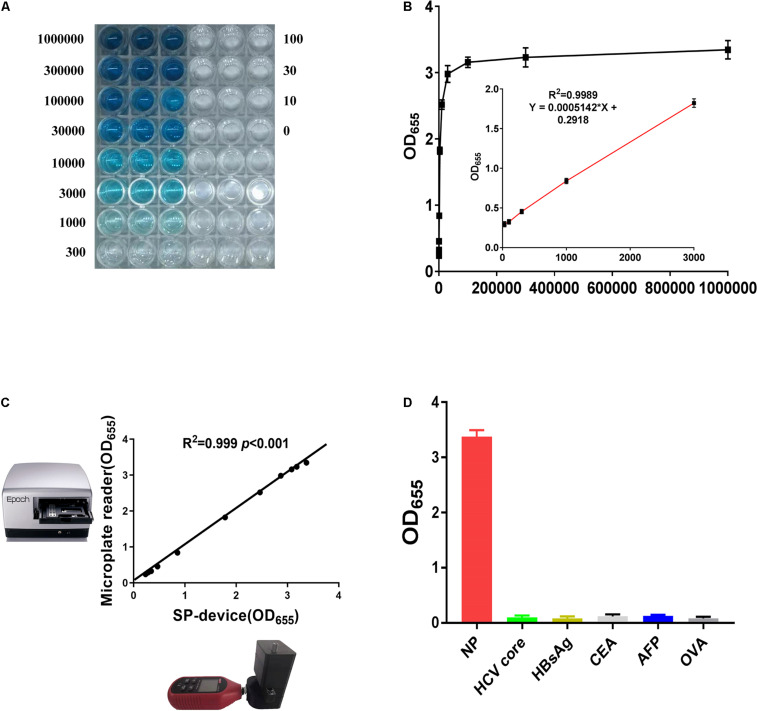
The sensitivity and specificity of SP-NLISA. **(A,B)** The standard curve and linear range of SP-NLICA for NP detection. **(C)** The results tested by SP-device and commercial microplate reader exhibited an excellent correlation and no significant difference (*R*^2^ = 0.999, *P* < 0.001). **(D)** The specificity of SP-NLISA for NP detection. The added protein concentration was 1 μg/mL.

To establish the standard curve, the NP reference standards at different concentrations from 10 pg/mL to 1 μg/mL were measured by SP-NLISA (Figure 4A). The optical density (OD_655_) was used for calculation and data statistics for this system. The OD algorithm is shown in Eq. 1, where *I* represents transmitted light intensity at 655 nm and *I*0 is the intensity of light before it entered the substrate solution. For this experiment, the *I*0 value is 139,100 LUX.

(1) OD=Ig(I0/I)

The light intensity through the substrate was provided by direct readout from the smartphone, and an OD value could be calculated through this equation. In total, 50 healthy blood donor samples were used as negative controls in SP-NLISA. The appropriate cut-off value of this system was set as a mean value of negative control plus 2 SD. The LOD was defined as the lowest level of NP standard which was tested higher than the cut-off value of this system (Wu et al., 2021). Thus, the LOD of this system for detecting the NP standard was 10 pg/mL and the linear range of SP-NLISA was 30 pg/mL–3 ng/mL (Figures 4A,B). To testify the stability of this SP-device, the results obtained by SP-device were also compared with those obtained by the commercial microplate reader (Figure 4C), which showed an excellent correlation (*R*^2^ = 0.999, *P* < 0.001). By comparing with SP-NLISA, the NP standards were measured by three control assays with Pt@AuNPs-ELISA (Supplementary Figure 8A), HRP-ELISA (Supplementary Figure 8B), and commercial ELISA kit (Supplementary Figure 8C), respectively. The data obtained from these four methods were presented in Supplementary Table 2. The results showed that SP-NLISA could satisfy the requirement of sensitivity and had the advantage of time-saving for POCT in clinical practice.

### Specificity and Accuracy of SP-NLISA

To identify the assay’s specificity, 450 blood donor samples were tested by SP-NLISA in comparison with conventional ELISA, of which none of them (0/450) was found reactive by both assays, suggesting that the specificity of SP-NLISA was 100%. Additionally, several serum samples, including hepatitis C virus (HCV) core protein, hepatitis B surface antigen (HBsAg), carcinoembryonic antigen (CEA), α-fetoprotein (AFP), and ovalbumin (OVA), were tested by way of an SP-NLISA (Figure 4D), and this showed no cross-reactivity with these antigens.

The accuracy of the SP-NLISA was evaluated by examining the recovery rate and CV from intra- or inter-assay. Different concentrations of NP antigen spiked in negative serum samples were measured by an SP-NLISA. The recovery rates were between 95 and 105%, and the CV was <5% (Supplementary Table 3). The accuracy of the portable device of the SP-NLISA was also compared with the traditional microplate reader (Supplementary Table 4), which showed that the maximum variation was <5%.

We also used three different models of smartphones to test for serum samples containing NP reference standards at different concentrations from 10 pg/mL to 1 μg/mL (Supplementary Table 5). The results were consistent between different smartphones, which suggested that this SP-NLISA could be used with different models of smartphones.

### Detection of Clinical Samples

**FIGURE 5:**
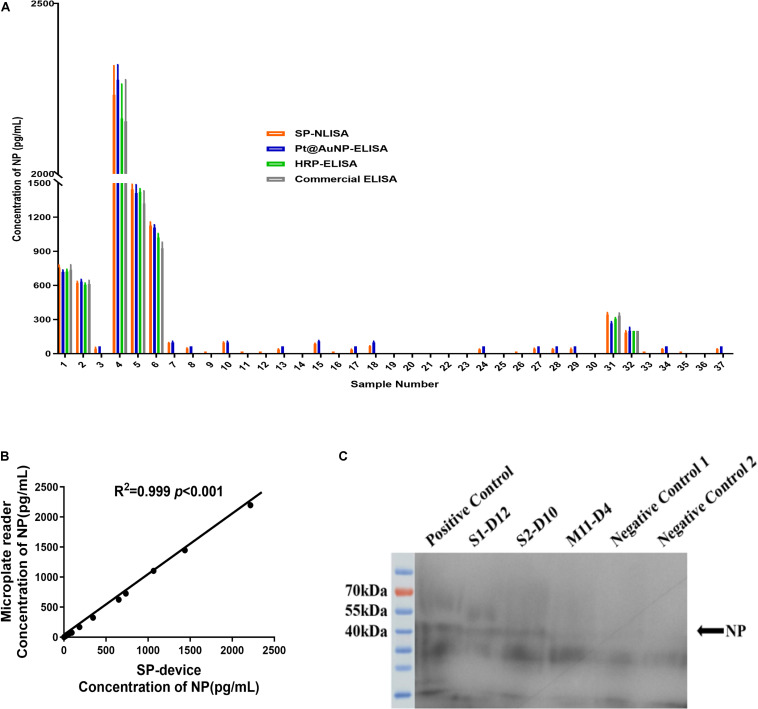
The clinical samples of COVID-19 patients were tested by SP-NLISA. **(A)** 37 clinical serum samples from 4 severe and 16 mild COVID-19 patients were tested for NP antigen of SARS-CoV-2 by SP-NLISA. The result was represented as a mean of triplicate for each sample. **(B)** Correlation between SP-device and commercial microplate reader was analyzed by SPSS 18.0, which exhibited an excellent correlation and no significant difference (*R*^2^ = 0.999, *P* < 0.001). **(C)** Identification of NP antigen in serum samples by Western blot. S1-D12, S2-D10, M11-D4 were COVID-19 patients’ samples collected at day 12, 10, or 4 post symptom onset. Positive control was a negative control sample with the addition of 1 μg/mL NP standard. Negative control 1 was the S4-D27 sample, while negative control 2 was a blood donor sample obtained from the Guangzhou blood center before the COVID-19 outbreak.

By using the established SP-NLISA, 37 clinical serum samples, including 11 index and 26 follow-up samples from 4 severe and 16 mild COVID-19 patients, were tested for NP antigens of SARS-CoV-2 (Figure 5A and Supplementary Table 6). Among 37 serum samples, 28 (75.7%) samples were detected as positive by SP-NLISA, while 21 (56.7%), 7 (18.9%), and 7 (18.9%) samples were found to be positive by use of a Pt@AuNPs-ELISA, HRP-ELISA, or commercial ELISA kit, respectively (Table 1). SP-NLISA had significantly higher sensitivity for detection of SARS-CoV-2 NP than those three ELISA methods (*P* < 0.001). The results obtained by SP-device were also compared with those obtained by the commercial microplate reader (Figure 5B), which exhibited an excellent correlation (*R*^2^ = 0.999, *P* < 0.001), indicating that this SP-NLISA was reliable and could be potentially used for POCT of SARS-CoV-2 infection.

**TABLE 1:** Sensitivity of SP-NLISA and control assays for testing of NP antigens in serums from COVID-19 patients.

Clinical typing	Sample (Nb)	SP-NLISA (Nb/%)	Pt@AuNP ELISA (Nb/%)	HRP-ELISA (Nb/%)	Biodragon ELISA (Nb/%)
Severe	14	13 (92.8%)	10 (71.4%)	5 (35.7%)	5 (35.7%)
Mild	23	15 (65.2%)	11 (47.8%)	2 (8.7%)	2 (8.7%)
Overall	37	28 (75.7%)	21 (56.7%)	7 (18.9%)	7 (18.9%)

Two samples from severe patients (S1 and S2) and a sample from a mild patient (M11) were detected positive by SP-NLISA, which were further identified by Western Blot analysis (Figure 5C), showing that a specific NP protein band in SP-NLISA positive serum samples but not in SP-NLISA negative or control samples. Thus, these results showed that SP-NLISA detection corresponded to the SARS-CoV-2 NP antigen.

## Discussion

The development of convenient and rapid detection methods for SARS-CoV-2 infection is important, as the appropriate treatment and isolation are hardly provided in a timely manner. From the perspective of accuracy, the NAT for SARS-CoV-2 RNA is the golden standard. However, this method requires a special testing platform and is time consuming. Thus, an alternative assay can facilitate the large-scale screening of populations, such as testing of viral antigen for the presence of virus in respiratory or blood samples.

As the throat swab samples were hard to collect from hospitals, we did not test antigens in the respiratory tract using this method. Compared with antigens in the respiratory tract collected by throat swab, the coronavirus antigens in the serum were effective supplementaries for sample collection and detection in terms of concentration and sustainability (Che et al., 2004; Li et al., 2005). In this study, 37 serum samples collected from 4 severe and 16 mild COVID-19 patients were tested for NP antigens by an SP-NLISA. An overall detection rate for NP antigens was 75.7%, of which 92.8% were from severe patients and 65.2% from mild patients (Table 1), which is higher than other reports (Supplementary Table 7; Hirotsu et al., 2020; Lambert-Niclot et al., 2020; Mak et al., 2020; Mertens et al., 2020; Scohy et al., 2020). The LOD of this system was as low as 10 pg/mL, while the LOD of conventional ELISA was usually above 100 pg/mL (Dinnes et al., 2020). In addition to the advantage of sensitivity, the testing could be carried out within 1 h and was thus time saving compared with several hours by conventional ELISA. This method was also cost-effective with a self-produced POCT device, which could be performed in biological safety hoods or cabinets for avoiding possible aerosol contamination, and the data could be transmitted *via* wifi to a wireless connecting mobile smart-phone for processing efficiently. This assay was easy to operate and thus did not need well-trained technicians and could be used for self-check at home or in grassroots hospitals. This assay could not only test SARS-CoV-2 antigens in serum but also detect other viral antigens. Thus we will try this assay for on-site testing of other infectious diseases and develop a new app for the smartphone to analyze the intensity of images of wells and to achieve the results more efficiently.

## Conclusion

In conclusion, we have developed a SP-NLISA, which could be carried out within 1 h using a portable detection platform that was easy to operate and to avoid possible aerosol contamination by reporting results *via* Bluetooth and could be effectively used in grassroots hospitals for on-site screening of SARS-CoV-2 infection with limited cost.

## Data Availability Statement

The original contributions presented in the study are included in the article/Supplementary Material, further inquiries can be directed to the corresponding author/s.

## Ethics Statement

The studies involving human participants were reviewed and approved by the Shenzhen Center for Disease Control and Prevention. Written informed consent to participate in this study was provided by the participants’ legal guardian/next of kin.

## Author Contributions

CL, XT, SH, and BL designed the study. BL, CLL, XT, ZW, and SH performed the experiments. JHL, BL, CL, and TL analyzed the data. JL, YF, and WZ provided the materials. BL, LZ, and CL wrote the manuscript. All authors contributed to the article and approved the submitted version.

## Conflict of Interest

The authors declare that the research was conducted in the absence of any commercial or financial relationships that could be construed as a potential conflict of interest.

## Publisher’s Note

All claims expressed in this article are solely those of the authors and do not necessarily represent those of their affiliated organizations, or those of the publisher, the editors and the reviewers. Any product that may be evaluated in this article, or claim that may be made by its manufacturer, is not guaranteed or endorsed by the publisher.
