# Supplementary material for: Development of a Smartphone-Based Nanozyme-Linked Immunosorbent Assay for Quantitative Detection of SARS-CoV-2 Nucleocapsid Phosphoprotein in Blood
Source: Front Microbiol. 2021 Aug 23;12:692831. doi: 10.3389/fmicb.2021.692831 (PMC8420716; doi:10.3389/fmicb.2021.692831)
Supplement: Supplementary file 1 [file Data_Sheet_1.DOCX]

Supplementary Material

**1. Characterization of Pt@AuNPs**

Pt@AuNPs have been reported to catalyze TMB and H_2_O_2_ efficiently (Liu et al., 2017). In this study, the ratios of 0.5%，1%，1.5%，2%，3%，4%，6% and 10% chloroplatinic acid (H_2_PtCl_6_) were compared for generating Pt@AuNPs (Supplementary Figure 4A), of which 6% and 10% testing group showed the best catalytic activity. The UV–vis spectra of AuNPs showed special optical absorption nearby 520 nm, while the generated Pt@AuNPs corresponding to 8 testing groups of H_2_PtCl_6_ lost the absorbance peak at 520 nm (Supplementary Figure 4B), indicating that the AuNPs were successfully converted to Pt@AuNPs and 6% H_2_PtCl_6_ were at the most suitable ratio. To analyze the catalytic properties of the generated Pt@AuNPs, and the catalytic effect was compared at different concentrations of HRP and Pt@AuNPs (2-50pM) in 0.1-10M H_2_O_2_ solution (Supplementary Figure 4C and 4D). The results showed that Pt@AuNPs appeared to be more catalytically active than HRP. The concentration of 25pM Pt@AuNPs in 1M H_2_O_2_ presented the optimal catalytic reaction.

**2. The conditions for catalytic reaction by Pt@AuNPs**

At the meantime, the additional conditions for catalytic reaction by Pt@AuNPs were optimized (Supplementary Figure 5). The sample buffer at pH8 was determined for most effective (Supplementary Figure 5A), and 10min reaction time was sufficient for reaching at a plateau (Supplementary Figure 5B). The temperature (0-80℃) and storage time (up to 30 days) did not influence the catalytic reaction of Pt@AuNPs but affected HRP activity (Supplementary Figure 5C and 5D), indicating that Pt@AuNPs had good stability and also high catalytic efficiency.

**3. The analysis of the three control assays**

The reference NP standards at different concentration from 10 pg/mL to 1 μg/mL were measured by these three control assays (Supplementary Figure 8) and the results were obtained by a commercial reader (Epoch) which was purchased from BioTek Instruments (Vermont, U.S.A.). The negative control and cut-off value of each assay was set as the same as SP-NLISA. The LOD was defined as the lowest level of NP standard which was tested higher than the cut-off value.

**4. The analysis of detection results of NP antigen in serum samples from COVID-19 patients by SP-NLISA**

Among 37 clinical serum samples, 14 serum samples were collected and detected in the follow-up from 4 severe COVID-19 patients (S1-S4) at 3-5 time-points during a month, which presented obviously higher level of SARS-CoV-2 NP than those 23 samples collected from 16 mild patients (M1-M16) (*P*<0.01, Supplementary Figure 9A). The concentration of NP antigen was found higher at early phase (1-10 days) but lower at late phase (21-30 days) during the course of disease post symptom onset (Supplementary Figure 9B). These results suggested that the NP antigen could persist for a month in blood of SARS-CoV-2 infected individuals with severe or mild symptoms.

# Supplementary Tables

| **Supplementary Table 1: The cost of each component of SP-NLISA device** | |
| --- | --- |
| **Material composition** | **Cost($)** |
| **3D printing materials** | **0.2** |
| **Laser source** | **0.5** |
| **Battery** | **0.4** |
| **Switch** | **0.1** |
| **Photometer** | **18** |
| **Adjustable resistence** | **0.3** |
| **USB charging** | **0.2** |
| **Others** | **0.3** |
| **Total** | **20** |

| **Supplementary Table 2: Comparison of different methods for NP testing** | | | | | |
| --- | --- | --- | --- | --- | --- |
| **Method** | **Limit of detection**  **(pg/mL)** | **Linear range**  **(pg/mL)** | **Time** | **Antibodies** | **Equipment** |
| SP-NLISA | 10 | 30-3000 | 1h | Self- produced | SP-device |
| Pt@AuNPs-  ELISA | 30 | 100-10000 | 2.5h | Self- produced | Microplate  reader |
| HRP-  ELISA | 100 | 300-30000 | 2.5h | Self- produced | Microplate  reader |
| Biodragon  ELISA | 100 | 300-30000 | 2.5h | commercial kit | Microplate  reader |

**Supplementary Table 3: Accuracy analysis for detection of NP antigen**

|  | **Spiking level (pg/ml)** | $\bar{\boldsymbol{x}}$**±SD** | **Recovery (%)** | **CV (%)** |
| --- | --- | --- | --- | --- |
| Intra assay(n=3) | 50 | 48.1±1.6 | 96.2 | 3.3 |
|  | 200 | 195.5±5.2 | 97.7 | 2.7 |
|  | 500 | 515±10.6 | 103 | 2.1 |
| Inter assay(n=3) | 50 | 47.8±2.1 | 95.6 | 4.4 |
|  | 200 | 192±6.8 | 96 | 3.5 |
|  | 500 | 519±15.5 | 103.8 | 2.9 |

Recovery (%) = (Mean of results tested by SP-NLISA /spiking level) *100%;

Coefficient of variation (CV) = (S.D. / Mean) *100%

| **Supplementary Table 4: Testing of NP standard by SP-NLISA device and microplate reader** | | | | |
| --- | --- | --- | --- | --- |
| **NP (pg/mL)** | ***I*(Lux)** | **Optical density (OD)** | | **Recovery (%)** |
|  |  | SP-device | Microplate reader |  |
| 1000000 | 63±8 | 3.346±0.055 | 3.371±0.084 | 99.2% |
| 300000 | 82±11 | 3.232±0.059 | 3.182±0.075 | 101.5% |
| 100000 | 97±7 | 3.157±0.031 | 3.081±0.066 | 102.4% |
| 30000 | 146±17 | 2.981±0.051 | 2.87±0.088 | 103.8% |
| 10000 | 421±28 | 2.52±0.029 | 2.463±0.078 | 102.3% |
| 3000 | 2092±104 | 1.823±0.022 | 1.783±0.077 | 102.3% |
| 1000 | 20141±629 | 0.839±0.014 | 0.854±0.049 | 98.2% |
| 300 | 48880±1252 | 0.454±0.012 | 0.466±0.026 | 97.4% |
| 100 | 65810±1733 | 0.325±0.011 | 0.341±0.027 | 95.3% |
| 30 | 70531±2111 | 0.295±0.013 | 0.287±0.022 | 102.8% |
| 10 | 72389±2250 | 0.284±0.014 | 0.276±0.016 | 102.9% |
| 0 | 80498±2651 | 0.238±0.014 | 0.232±0.011 | 102.6% |

The results were shown as $\bar{x}$ ± SD. $\bar{x}$: Average (n=3); S.D.: Standard deviation (n=3). The OD values were calculated by the equation: OD=log(*I*0/*I*), *I*0=139100 lux. Recovery (%) = (Results of SP-device / Results of microplate reader) *100%

**Supplementary Table 5: NP reference standards tested by different smartphones**

| Sample  Model | | 1 | 2 | 3 |
| --- | --- | --- | --- | --- |
| Huawei  Honor 10 | *I*(mean) | 72115 | 64277 | 19829 |
|  | OD | 0.285 | 0.335 | 0.846 |
| iPhone 8 | *I*(mean) | 53422 | 47897 | 14748 |
|  | OD | 0.283 | 0.331 | 0.842 |
| Vivo X7 | *I*(mean) | 39603 | 35292 | 10881 |
|  | OD | 0.282 | 0.332 | 0.843 |

The corresponding *I*0 values of the three smartphones were adjusted to 139100, 102500 and 75800 lux, respectively, while concentrations of NP reference standards corresponding to sample 1, 2 and 3 were 10 pg/mL, 100 pg/mL and 1 μg/mL, respectively.

| **Supplementary Table 6: Detection of NP antigen in serum samples of COVID-19 patients by SP-NLISA and control assays** | | | | | | | | | | |
| --- | --- | --- | --- | --- | --- | --- | --- | --- | --- | --- |
| **Sample No.** | **Patient No.** | **Clinical typing** | **Age** | **Days post symptom onset** | **NP (pg/mL)** | | | | | |
|  |  |  |  |  | **SP-NLISA** | **Pt@AuNP**  **ELISA** | | **HRP ELISA** | **Commercial ELISA** | |
| 1 | S1 | severe | 50 | 12 | 735 ±10 | 724 ±29 | 736 ±49 | | 739 ±46 |  |
| 2 |  |  |  | 19 | 650±10 | 632±19 | 622±37 | | 635±18 |  |
| 3 |  |  |  | 26 | 43±12 | + | - | | - |  |
| 4 | S2 | severe | 65 | 10 | 2216±18 | 2257±45 | 2112 ±134 | | 2189 ±65 |  |
| 5 |  |  |  | 18 | 1437±85 | 1392±84 | 1225±112 | | 1348±101 |  |
| 6 |  |  |  | 22 | 1064±88 | 1016±21 | 1037±85 | | 1055±84 |  |
| 7 | S3 | severe | 67 | 16 | 93±9 | 105±5 | - | | - |  |
| 8 |  |  |  | 20 | 44±6 | + | - | | - |  |
| 9 |  |  |  | 25 | + | - | - | | - |  |
| 10 | S4 | severe | 55 | 12 | 79±9 | 110±10 | - | | - |  |
| 11 |  |  |  | 14 | + | - | - | | - |  |
| 12 |  |  |  | 19 | + | - | - | | - |  |
| 13 |  |  |  | 22 | 37±6 | + | - | | - |  |
| 14 |  |  |  | 27 | - | - | - | | - |  |
| 15 | M1 | mild | 76 | 15 | 77±6 | 107±8 | - | | - |  |
| 16 |  |  |  | 19 | + | - | - | | - |  |
| 17 |  |  |  | 24 | 35±5 | + | - | | - |  |
| 18 | M2 | mild | 68 | 13 | 58±8 | + | - | | - |  |
| 19 |  |  |  | 20 | - | - | - | | - |  |
| 20 | M3 | mild | 70 | 10 | - | - | - | | - |  |
| 21 | M4 | mild | 57 | 12 | - | - | - | | - |  |
| 22 |  |  |  | 16 | - | - | - | | - |  |
| 23 |  |  |  | 28 | - | - | - | | - |  |
| 24 | M5 | mild | 56 | 12 | 36±4 | + | - | | - |  |
| 25 | M6 | mild | 60 | 19 | - | - | - | | - |  |
| 26 | M7 | mild | 7 | 1 | + | - | - | | - |  |
| 27 | M8 | mild | 1 | 7 | 42±6 | + | - | | - |  |
| 28 | M9 | mild | 33 | 9 | 39±5 | + | - | | - |  |
| 29 |  |  |  | 13 | 39±8 | + | - | | - |  |
| 30 | M10 | mild | 57 | 20 | - | - | - | | - |  |
| 31 | M11 | mild | 27 | 4 | 345±17 | 345±25 | 314±27 | | 324±26 |  |
| 32 | M12 | mild | 46 | 5 | 184±17 | 200±21 | + | | + |  |
| 33 | M13 | mild | 25 | 9 | + | - | - | | - |  |
| 34 | M14 | mild | 36 | 8 | 40±4 | + | - | | - |  |
| 35 | M15 | mild | 21 | 4 | + | - | - | | - |  |
| 36 |  |  |  | 12 | - | - | - | | - |  |
| 37 | M16 | mild | 20 | 8 | 41±2 | + | - | | - |  |

The concentrations of NP antigen in serum samples were shown as $\bar{x}$±SD. $\bar{x}$: Average of NP antigen concentrations (n=3); S.D.: Standard deviation. The symbol of "+" means the sample is positive for NP testing but the concentration of NP in the sample is lower than the detection limit of the linear range.

| **Supplementary Table 7: Previous reports for NP testing** | | | | |
| --- | --- | --- | --- | --- |
| **Article** | **Method** | **Sensitivity** | **Specificity** | **Sample** |
| Mertens et al., 2020 | colloidal gold based strip | 57.6% | 99.5% | nasopharyngeal  swab |
| Hirotsu, et al., 2020 | chemiluminescence enzyme immunoassay | 55.2% | 99.6% | nasopharyngeal swab |
| Lambert-Niclot, et al., 2020 | colloidal gold based strip | 50% | 100% | nasopharyngeal swab |
| Mak, et al., 2020 | colloidal gold based strip | 11.1% | 100% | sputum |
| Scohy, et al., 2020 | colloidal gold based strip | 30.2% | 100% | nasopharyngeal swab |
| **Our method** | NLISA | 75.7% | 100% | serum |

# Supplementary Figures

**
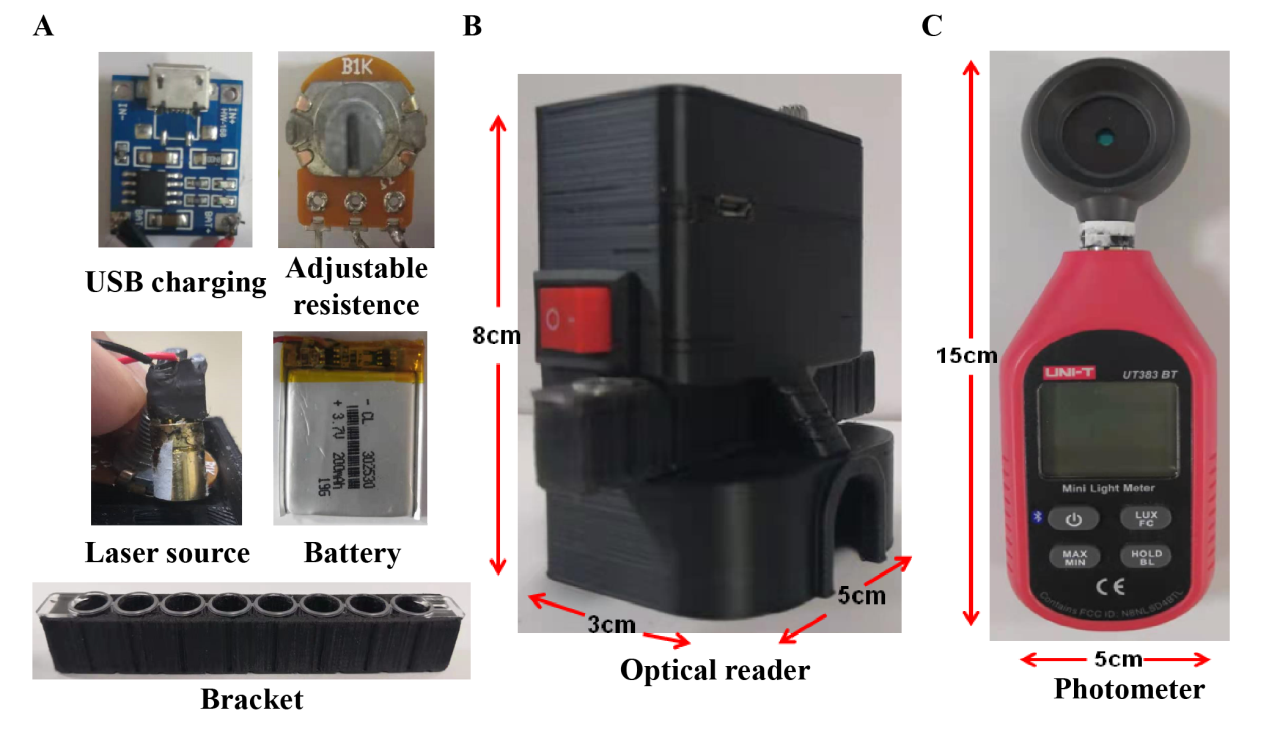
**

**Supplementary Figure 1: The complete structure and material composition of SP-device.** (A) The elements of device is mainly composed of USB charging, adjustable resistance, laser source and battery; (B) The size of this device; (C) The photometer used for SP-device.


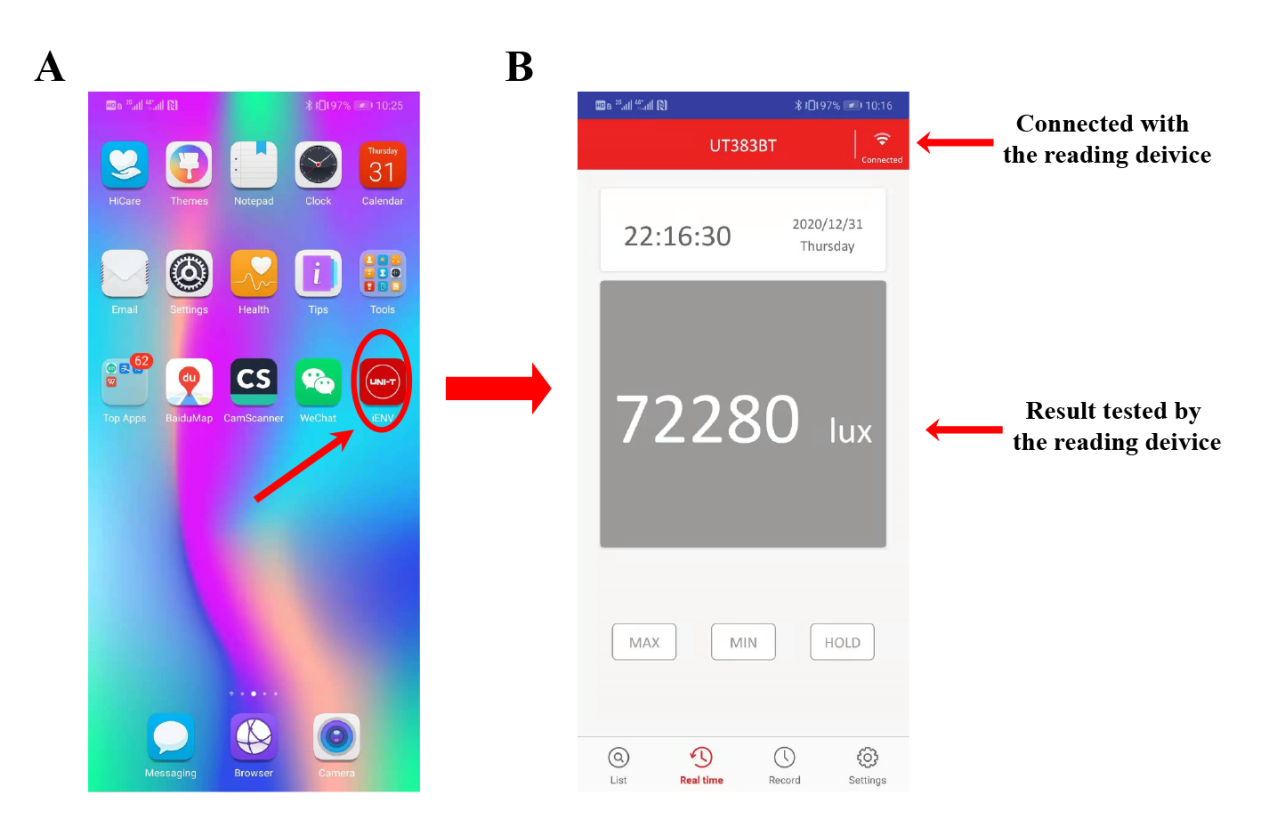


**Supplementary Figure 2: Instructions for using the iENV app.** (A) The icon of iENV on the phone desktop. (B) The principal sheet of iENV. When the device for SP-NLISA was connected with iENV through Bluetooth, the results tested by this device was shown in iENV.


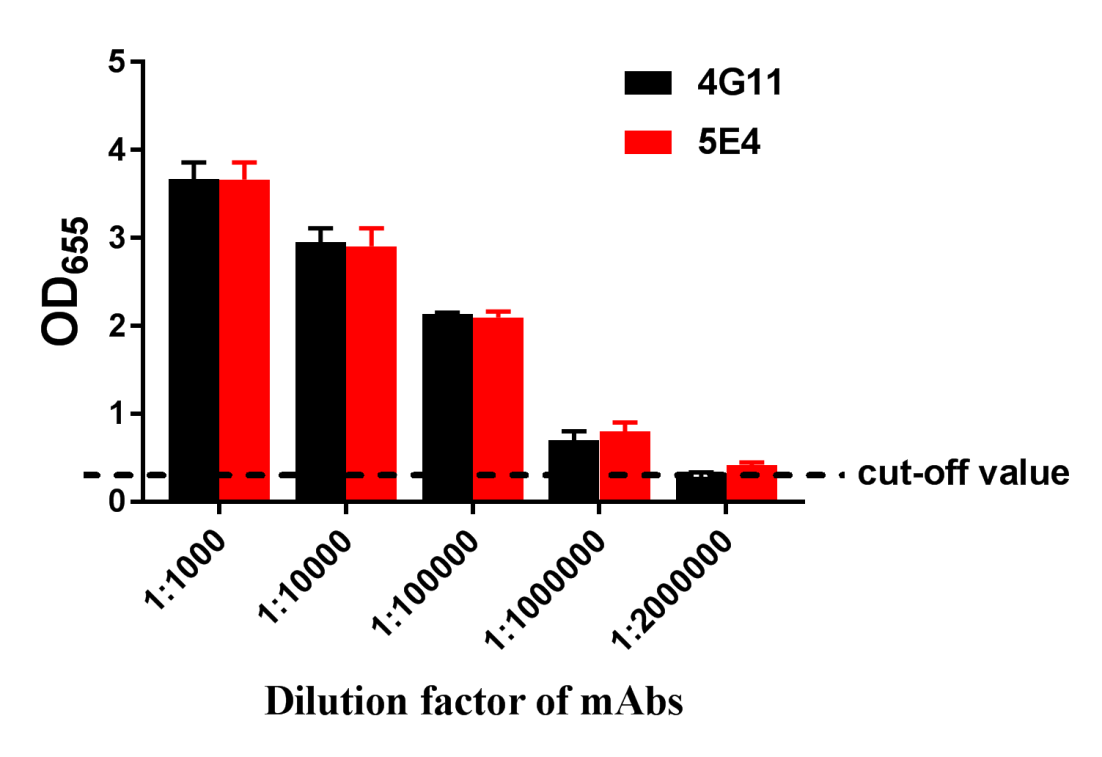


**Supplementary Figure 3:** The titers of the purified ascites fluids (clone 4G11 and clone 5E4) by ELISA. Fifty healthy blood donor plasma samples were tested and set as negative control. The cut-off value was set as a mean value of negative control plus 2 SD.


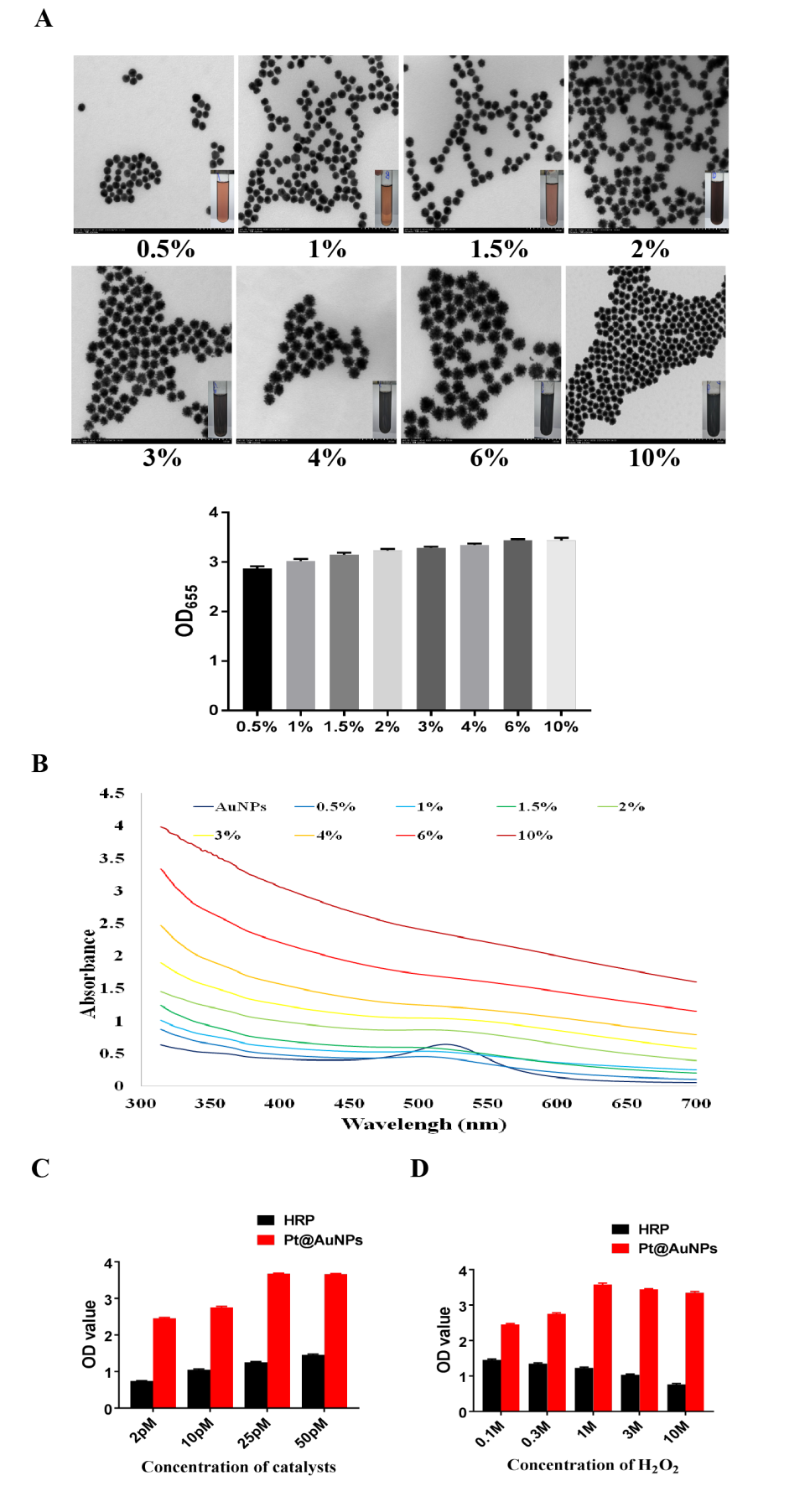


**Supplementary Figure 4: Characteristic catalytic effects of Pt@AuNPs.** (A) The catalytic effects of synthesized Pt@AuNPs with different sizes and percentages. (B) The UV–vis spectra of AuNPs and Pt@AuNPs. (C) The catalytic effect of different concentration of HRP and Pt@AuNPs in 10 M H_2_O_2_. (D) The catalytic performance of HRP and Pt@AuNPs at different concentration of H_2_O_2_.


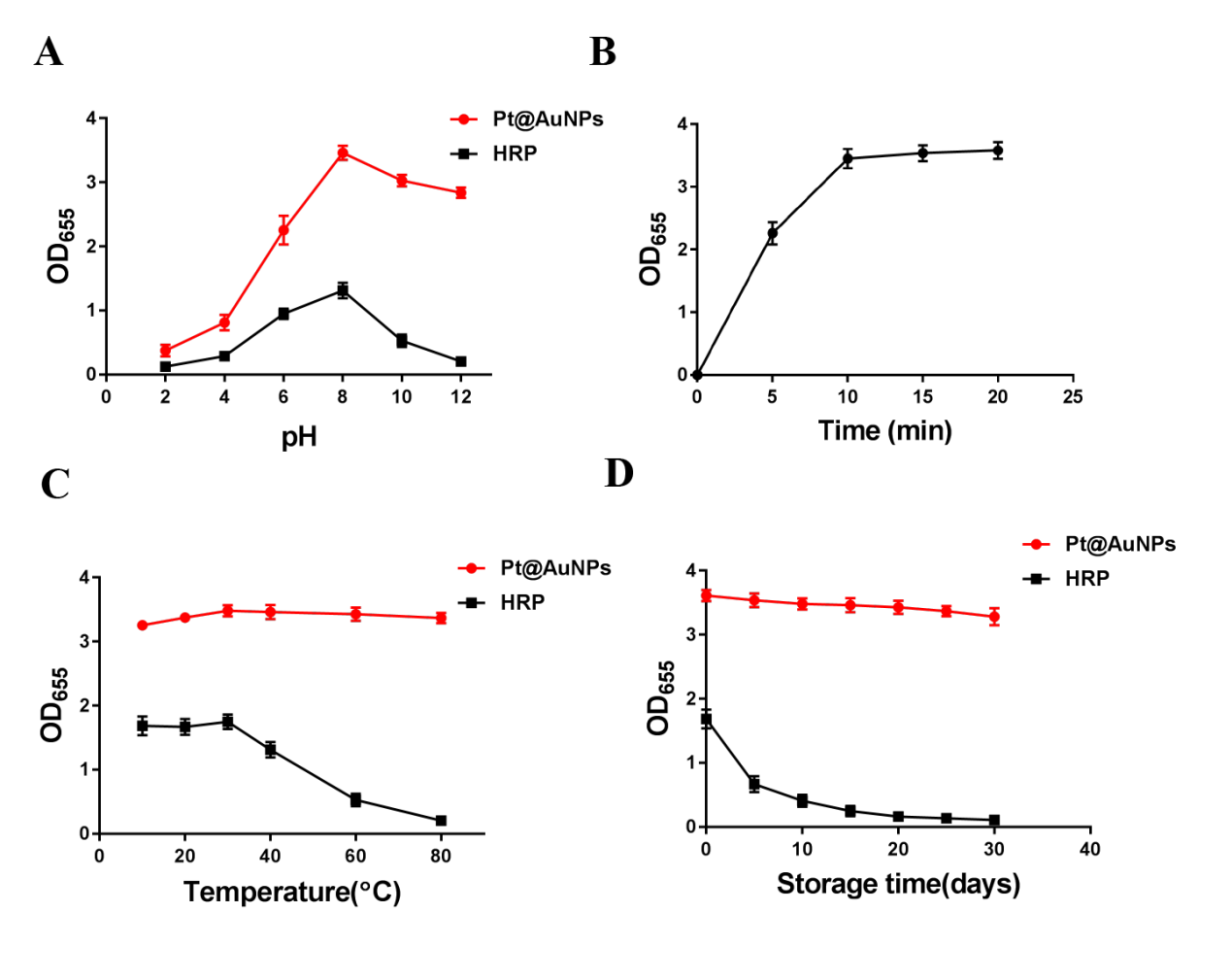


**Supplementary Figure 5: The stability of the synthesized Pt@AuNPs.** (A) The activities of HRP and Pt@AuNPs at different pH values. (B) The time curve of the catalytic reaction for Pt@AuNPs. (C-D) The activities of HRP and Pt@AuNPs at different times and temperatures.


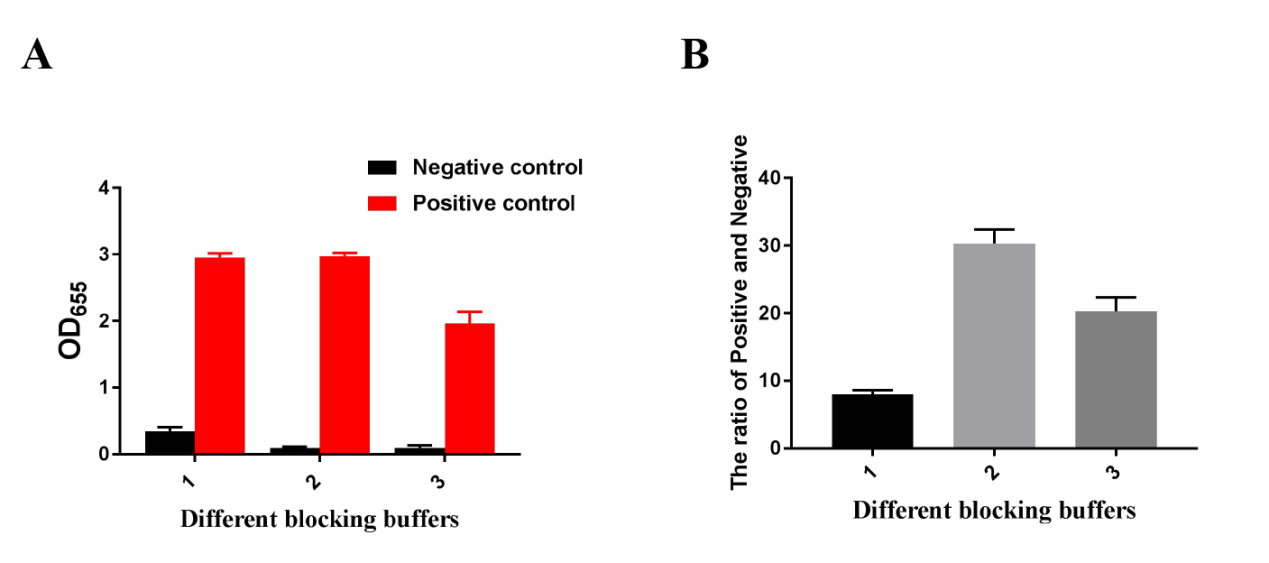


**Supplementary Figure 6: Optimization of the blocking buffer for mAb1-Pt@AuNPs.** (A) The OD values of different blocking buffers: 1. PBST +1% BSA; 2. PBST +1% BSA + 0.1% casein; 3. PBST +1% BSA + 1% casein. (B) Ratio of positive and negative samples. The negative control was a sample from a healthy blood donor and the positive control was a negative control sample with the addition of 1 μg/mL NP standard.


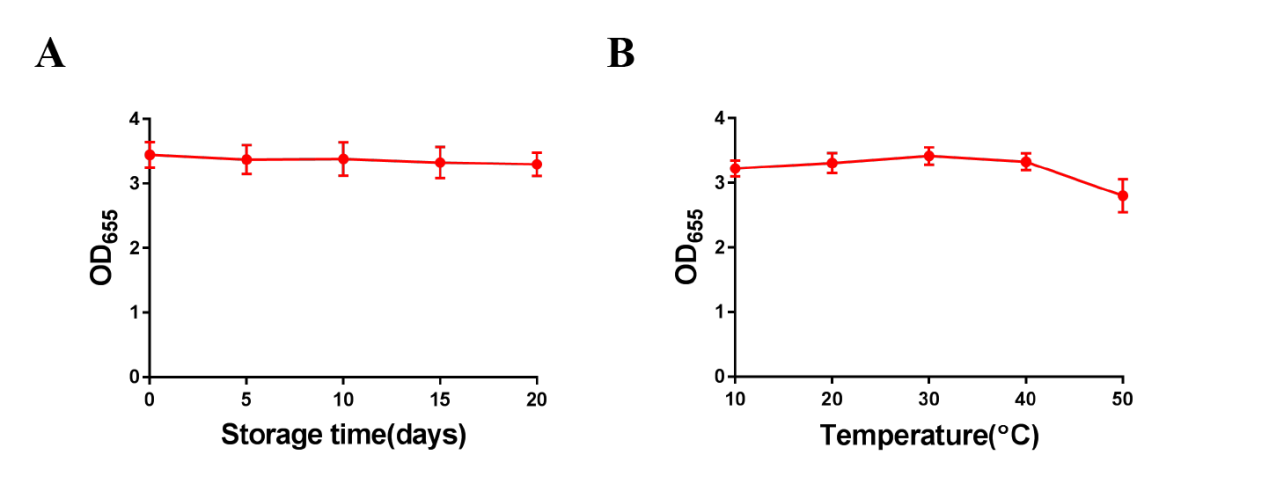


**Supplementary Figure 7:**  **The stability of SP-NLISA.** (A) The activity of SP-NLISA at different storage times at 37℃, or (B) at different temperatures.


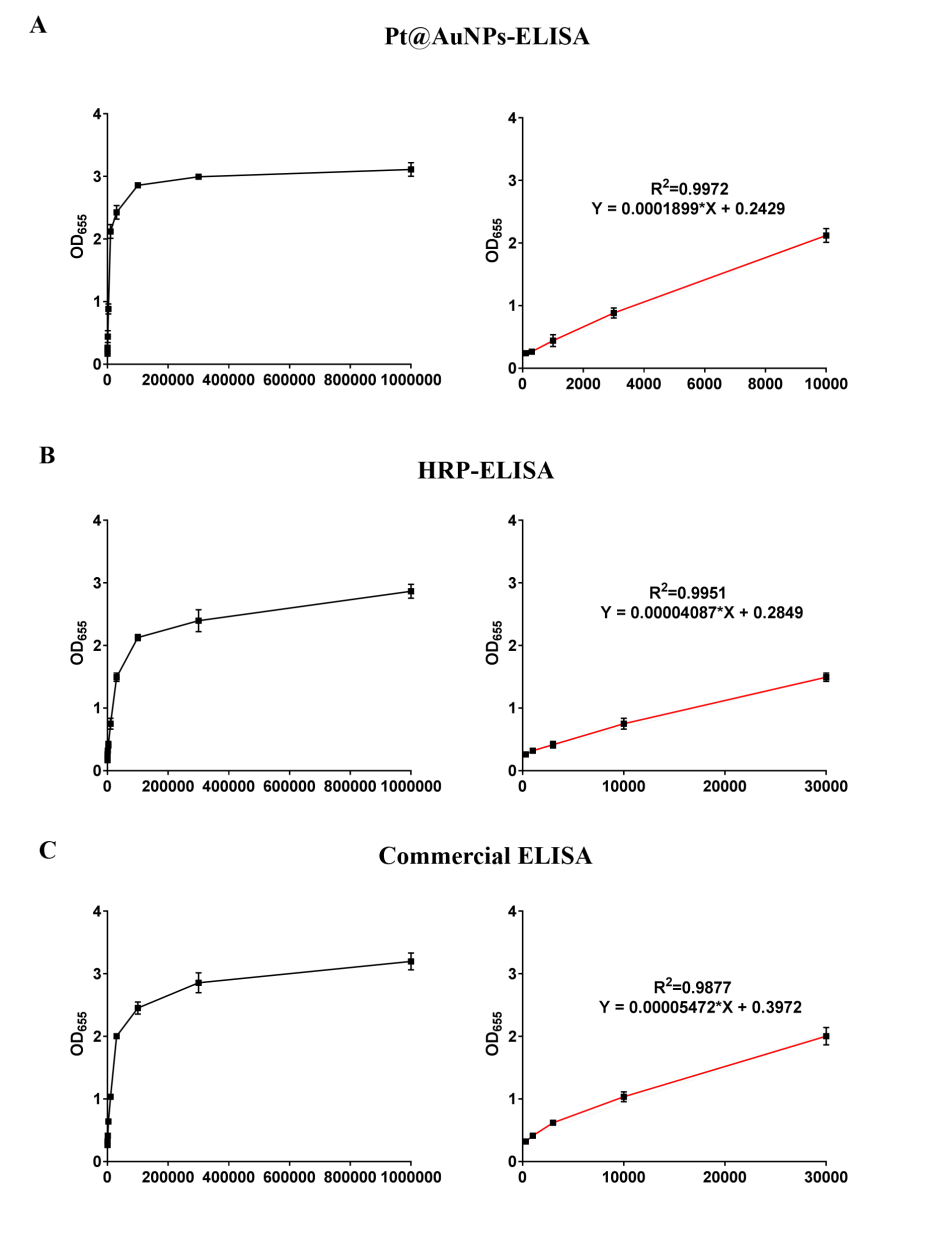


**Supplementary Figure 8: The standard curve and linear range for measurement of NP detection by control assays.** (A) Pt@AuNPs-ELISA, (B) HRP-ELISA and (C) a commercial ELISA kit.


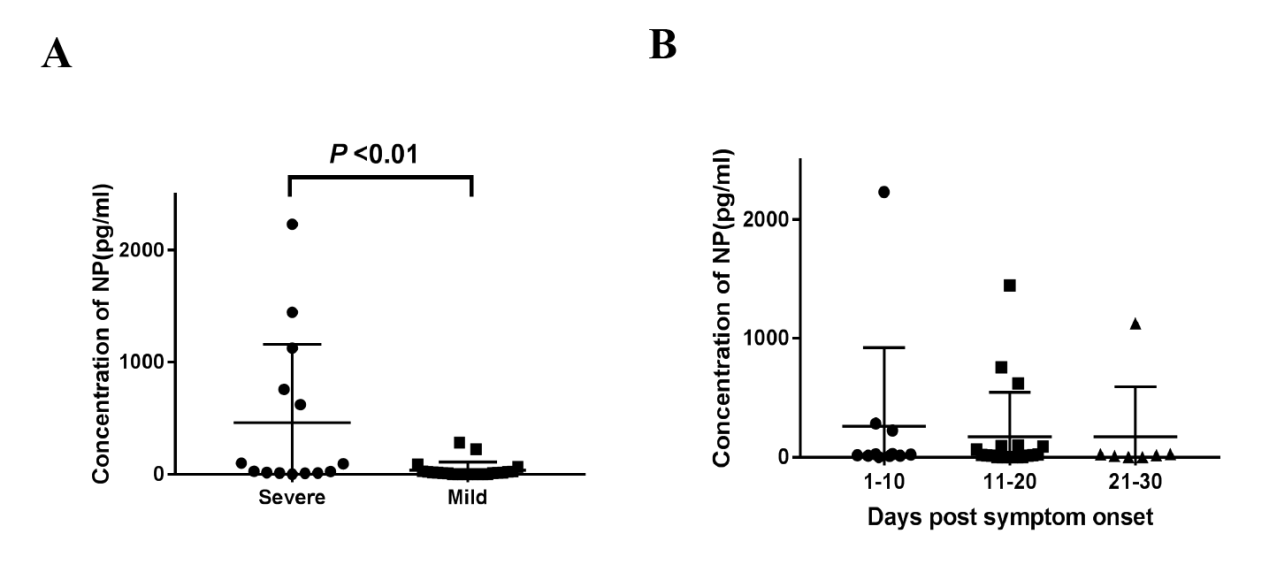


**Supplementary Figure 9: Clinical detection of NP in serum samples from COVID-19 patients by SP-NLISA. The samples were stratified according to patients’ symptoms or days post symptom onset.** (A) Detection for NP in serum samples from severe or mild COVID-19 patients. (B) Detection for NP in serum samples collected on the days post symptom onset of patients.
